# Genome-Based Characterization of *Listeria monocytogenes*, Costa Rica

**DOI:** 10.3201/eid2912.230774

**Published:** 2023-12

**Authors:** María Giralt-Zúñiga, Mauricio Redondo-Solano, Alexandra Moura, Nathalie Tessaud-Rita, Hélène Bracq-Dieye, Guillaume Vales, Pierre Thouvenot, Alexandre Leclercq, Carolina Chaves-Ulate, Kattia Núñez-Montero, Rossy Guillén-Watson, Olga Rivas-Solano, Grettel Chanto-Chacón, Francisco Duarte-Martínez, Vanessa Soto-Blanco, Javier Pizarro-Cerdá, Marc Lecuit

**Affiliations:** Institut Pasteur, Université Paris Cité, Inserm U1117, Biology of Infection Unit, National Reference Center, and WHO Collaborating Center *Listeria*, Paris, France (M. Giralt-Zúñiga, M. Redondo-Solano, A. Moura, N. Tessaud-Rita, H. Bracq-Dieye, G. Vales, P. Thouvenot, A. Leclercq, J. Pizarro-Cerdá, M. Lecuit);; Instituto Tecnológico de Costa Rica, Cartago, Costa Rica (M. Giralt-Zúñiga, K. Núñez-Montero, R. Guillén-Watson, O. Rivas-Solano);; University of Costa Rica, San José, Costa Rica (M. Redondo-Solano, C. Chaves-Ulate);; Instituto Costarricense de Investigación y Enseñanza en Nutrición y Salud, Tres Rios, Costa Rica (G. Chanto-Chacón, F. Duarte-Martínez);; National Animal Health Service, Heredia, Costa Rica (V. Soto-Blanco);; Necker Enfants Malades University Hospital, APHP, Institut Imlagine, Paris (M. Lecuit)

**Keywords:** Listeria monocytogenes, bacteria, food safety, whole-genome sequencing, pathogen surveillance, outbreak detection, Costa Rica

## Abstract

Genomic data on the foodborne pathogen *Listeria monocytogenes* from Central America are scarce. We analyzed 92 isolates collected during 2009–2019 from different regions in Costa Rica, compared those to publicly available genomes, and identified unrecognized outbreaks. Our findings suggest mandatory reporting of listeriosis in Costa Rica would improve pathogen surveillance.

*Listeria monocytogenes* is a gram-positive pathogen responsible for listeriosis, a severe foodborne infection that causes high hospitalization and mortality rates in at-risk populations, including older adults, immunocompromised persons, pregnant women, and newborns (*1*). *L. monocytogenes* diversity can be classified into lineages, genoserogroups, clonal complexes (CCs), and sequence types (STs), defined by multilocus sequence typing (MLST) (*2*). Core-genome MLST (cgMLST) further identifies sublineages (SLs) and cgMLST types (CTs) (*2*). Major CCs and SLs are distributed globally and can be heterogeneous in terms of virulence; isolates from serogroup IVb (lineage I) often cause the most severe infections (*2**–**4*).

Pathogen surveillance using whole-genome sequencing (WGS) provides unprecedented resolution for identifying case clusters and contamination sources and for predicting strain virulence and antimicrobial resistance, which can aid in risk assessment (*2*,*5*). Previous studies confirmed *L. monocytogenes* in various foods in Costa Rica; reported contamination levels were 5%–20% in processed meat products and fresh cheeses (*6*,*7*). Because listeriosis is not a notifiable disease in Costa Rica, its prevalence is unknown, and diversity of *L. monocytogenes* circulating in the country is unclear.

To clarify the diversity of and potential public health risk from circulating strains, we used WGS to characterize 92 isolates recovered during 2009–2019 from 16 clinical, 67 food, and 9 production environment samples in Costa Rica (Appendix). When location data were available, isolates were from urban areas, including the capital city San José, and from rural areas where fresh cheese production is prevalent, including Alajuela, Naranjo, San Ramón, Vara Blanca, Upala, and Turrialba. Turrialba region accounts for 70% of fresh cheese produced in Costa Rica (Figure; Appendix).

**Figure:**
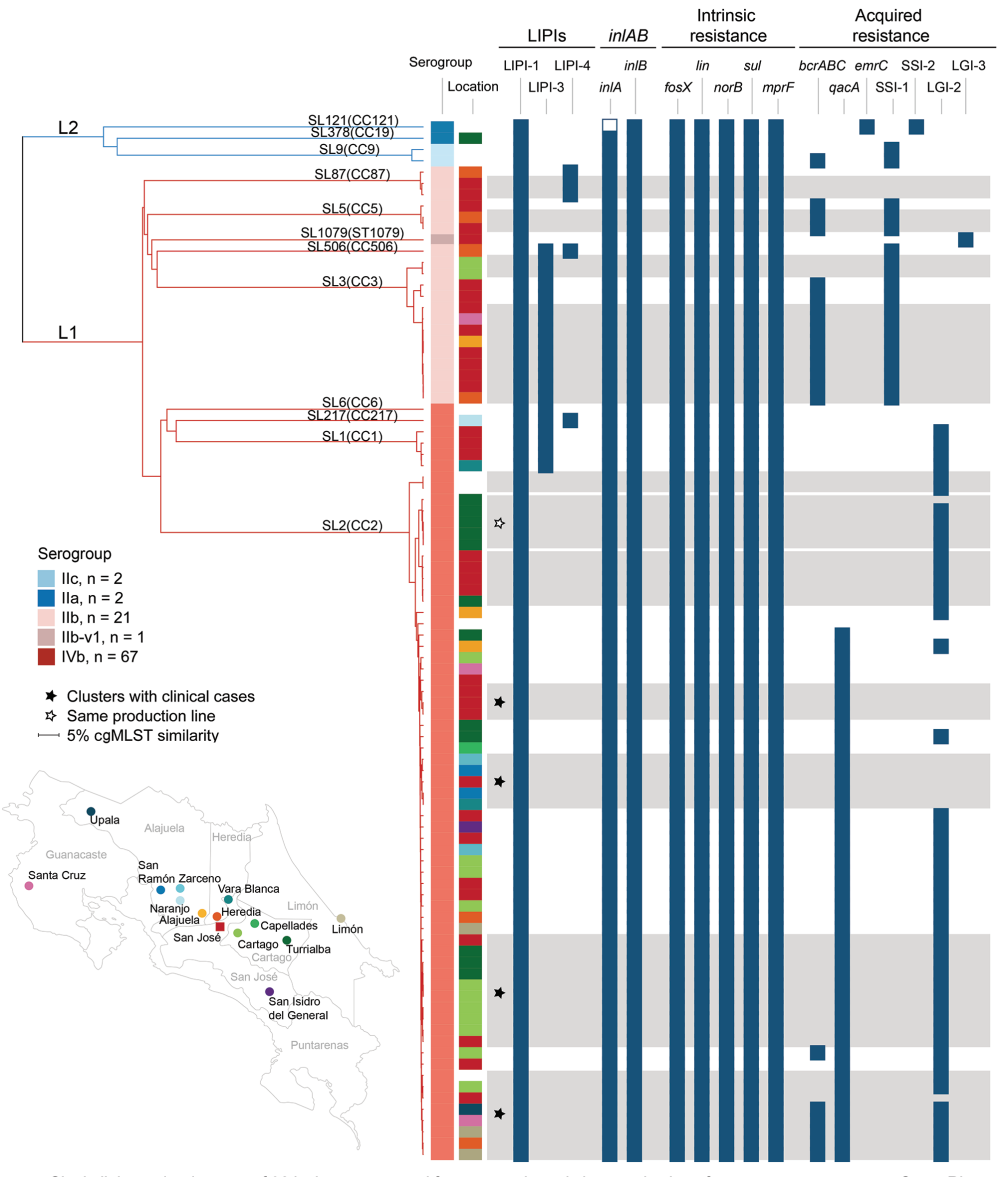
Single linkage dendrogram of 92 isolates generated for genome-based characterization of *Listeria monocytogenes*, Costa Rica. Dendrogram is based on core-genome multilocus sequence typing; (cgMLST) allelic profiles (1,748-locus scheme). Branches are colored according to lineages: L1, red; L2, blue. Branches are labeled according to lineages, sublineages, and clonal complexes. Information on isolates’ serogroup, and resistance profiles are provided in the columns. Colors in location column correspond to dots on map. Gray bars indicate clusters of isolates with <7 allelic differences out of 1,748 cgMLST loci. Presence of selected virulence and resistance genetic traits in each isolate is represented by dark blue boxes and empty boxes denote genes with premature stop codons. More details are provided in Appendix Figure 1. CC, clonal complex; L, lineage; LIPI, listeria pathogenicity island; SL, sublineage.

We found that isolates from lineage I (95%, n = 88) and lineage II (5%, n = 4) were unevenly distributed into 12 different SLs and CCs (Figure; Appendix Figure 1). Those isolates included a new lineage I sublineage, designated SL1079 (new MLST singleton ST1079), which was identified in an isolate from shrimp (cgMLST type L1-SL1079-ST1079-CT1669). That isolate had an atypical genoserogroup IIb profile, designated IIb-v1, that differed from the classic IIb profile by the presence of *lmo0737*. WGS confirmed the presence of *lmo0737* and flanking genes *lmo0733–39*, typically found in lineage II isolates from serogroups IIa and IIc but only occasionally found in lineage I serogroup IVb-v1 (*8*). Of note, 80% of isolates investigated from both clinical and food-associated sources were from sublineages SL2/CC2 (66%, n = 61) and SL3/CC3 (14%, n = 13). SL2/CC2 (serogroup IVb) and SL3/CC3 (serogroup IIb) isolates are found worldwide and are associated with invasive infections (*2*–*4*). However, they are rarely the most prevalent genotypes (*2**,**3*). Available data from other countries in Central America confirmed overrepresentation of SL2/CC2 and SL3/CC3 in Costa Rica (Appendix), which could be related to country’s geographic location, climatic peculiarities, commercial trends, or natural reservoirs.

At the strain level, we identified 48 CTs, of which 44 (92%) were not previouly reported. Eleven (23%) CTs included multiple isolates at a cutoff of 7 allelic differences of 1,748 cgMLST loci (*2*) (Table; Figure; Appendix Figures 1–3). Eight isolates were cgMLST type L1-SL2-ST2-CT2715, which accounted for 25% of clinical cases and spanned 9 years (Table).

**Table:** Sequence types identified in a genome-based characterization of *Listeria monocytogenes*, Costa Rica*

cgMLST type	CC	Serogroup	No. isolates (%)			Food type	Isolation years	Genetic resistance traits
			Total, n = 92	Clinical, n = 16	Nonclinical, n = 76			
L1-SL2-ST2-CT2715	CC2	IVb	8 (9)	4 (25)	4 (5)	Dairy, meat	2009, 2011, 2013, 2016–2017	*bcrABC*,* qacA,* LGI-2
L1-SL2-ST2-CT6120	CC2	IVb	10 (11)	2 (13)	8 (9)	Dairy	2010, 2013, 2016, 2018–2019	*qacA*, LGI-2
L1-SL2-ST2-CT2718	CC2	IVb	5 (5)	1 (6)	4 (5)	Dairy	2016, 2019	*qacA*
L1-SL2-ST1251-CT2780	CC2	IVb	3 (3)	1 (6)	2 (3)	Meat	2015–2016, 2018	*qacA*
L1-SL3-ST3-CT2730	CC3	IIb	9 (10)	0	9 (10)	Fish, meat	2016	*bcrABC*, SSI-1
L1-SL2-ST2-CT6072	CC2	IVb	5 (5)	0	5 (7)	Dairy	2019	LGI-2
L1-SL2-ST1627-CT6041	CC2	IVb	5 (5)	0	5 (7)	Dairy	2018–2019	LGI-2
L1-SL87-ST847-CT65	CC87	IIb	2 (2)	0	2 (3)	Meat	2016, 2019	NA
L1-SL3-ST1262/ST2762-CT2781	CC3	IIb	2 (2)	0	2 (3)	Dairy	2013	SSI-1
L1-SL5-ST5-CT2793	CC5	IIb	2 (2)	0	2 (3)	Fish, meat	2016	*bcrABC*, SSI-1
L1-SL2-ST2-CT2762	CC2	IVb	2 (2)	0	2 (3)	Mushrooms	2011	LGI-2

*Table includes types detected in this study comprising >2 isolates with a cutoff of 7 allelic differences, n = 11/48. CC, clonal complex; cgMLST, core-genome multilocus sequence typing; LGI, *Listeria* genomic island; NA, not applicable; SSI, stress survival islet.

Most human cases were associated with dairy products (Table). However, tracing to confirm the source of infection was not possible because most production is conducted by local farmers, often without traceability or attribution to the site of production.

Fresh cheese production is an economic staple in Costa Rica, and previous studies have reported *L. monocytogenes* detection in those products (*7*). Results from this study also show detection of identical strains of cgMLST type L1-SL2-ST2-CT6072 along the same production line, from raw materials to the final product, suggesting inadequate sanitation contributes to contamination (*9*). 

*L. monocytogenes* is problematic for the food industry because it can survive and multiply under adverse environmental conditions (*10*). In this study, 90% of isolates carried >1 genetic element encoding for tolerance to disinfectants or stress. Markers of tolerance to disinfectants included *qacA* (51%, n = 47), *bcrABC* (23%, n = 21), and *emrC* (1%, n = 1). In addition, isolates had stress survival islet (SSI) genes, including SSI-1, conveying tolerance to low pH and high salt concentrations (21%, n = 19), and SSI-2 conveying, tolerance to high pH and oxidative stress (1%, n = 1), as well as *Listeria* genomic island (LGI) genes, including LGI-2 (50%, n = 48) and LGI-3 (1%, n = 1) conveying tolerance to metals. Those tolerances can make *L. monocytogenes* elimination from production sites more difficult.

This study provides insight into the diversity of *L. monocytogenes* strains circulating in Central America and can aid national reference institutions in promoting regulatory changes to guarantee mandatory listeriosis reporting. In addition, institutions should establish mechanisms to provide low-cost microbiologic analysis. We also recommend regular sampling of risk products and training of artisanal processors. 

In conclusion, strengthened WGS surveillance in Costa Rica could assist in controlling *L. monocytogenes* and provide food producers with information on strain diversity and effective means of eradication. WGS surveillance also would enable authorities to detect outbreaks and trace sources of contamination.

Appendix Additional information on genome-based characterization of *Listeria monocytogenes*, Costa Rica.
