# Supplementary material for: Genome-Based Characterization of Listeria monocytogenes, Costa Rica
Source: Emerg Infect Dis. 2023 Dec;29(12):2566–9. doi: 10.3201/eid2912.230774 (PMC10683821; doi:10.3201/eid2912.230774)
Supplement: Appendix — Additional information on genome-based characterization of Listeria monocytogenes, Costa Rica. [file 23-0774-Techapp-s1.pdf]

Article DOI: https://doi.org/10.3201/eid2912.230774

EID cannot ensure accessibility for supplementary materials supplied by authors. Readers who have difficulty accessing supplementary content should contact the authors for assistance.

Genome-Based Characterization of *Listeria monocytogenes,* Costa Rica

Appendix

Materials and Methods

Bacterial Isolation

The study included 92 *L. monocytogenes* isolates previously collected from different regions throughout Costa Rica (Figure in main manuscript; Appendix Table 1) and spanning eleven years (2009–2019). Clinical isolates (n = 16) were obtained by the Institute for Research and Teaching in Nutrition and Health (INCIENSA) in the context of the activities of the Costa Rican National Clinical Laboratory Network, comprised by laboratories of major hospitals, clinical settings, and the University of Costa Rica (UCR). Even though *L. monocytogenes* is not a notifiable disease in the country, there is an established criterium where bacterial pathogens that cause meningitis are sent to INCIENSA for confirmation of identification as well as antibiotic susceptibility tests. Isolates from food and food-production environments (n = 76) were obtained by the National Laboratory of Veterinary Services (LANASEVE) of National Animal Health Service (SENASA), which performs microbiology analysis for the surveillance of food safety in products of animal origin for human consumption, by the INCIENSA that also monitors the microbiological quality of food for human consumption, and by the UCR and the Instituto Tecnológico de Costa Rica (ITCR) in the scope of research projects and/or routine analyses for customers, as well as in the framework of a specific study to investigate *Listeria* spp. strains previously isolated by private entities regulated and accredited by the international standard INTE/ISO/IEC 17025:2017 for microbiological analysis that guarantee the results of their sampling process.

Bacterial isolation was performed as described previously, following either the Bacteriological Analytical Manual method for *Listeria* isolation (*1*), for isolates obtained by the UCR, ITCR and SENASA, or the ISO 11290–1:2017 method (*2*), for isolates obtained by the INCIENSA.

Isolate identification was performed by proteomic analysis by matrix-assisted laser desorption/ionization time-of-flight (MALDI-TOF) mass spectrometry, using the MicroFlex LT system with MBT library DB-5989 (Bruker Daltonics, Bremen, Germany), as previously described (*3*).

DNA Extraction and Genome Sequencing

Isolates were cultured in Brain Heart Infusion Broth (BHI, Oxoid, Basingstoke UK) at 35°C overnight before use. DNA extraction was performed with the DNeasy Blood & Tissue Kit (QIAGEN, København Ø, Denmark-confirm), according to the instructions provided by the manufacturer. Qubit fluorometer (Thermo FisherScientific, Waltham, MA, USA) was used to evaluate DNA quantity and purity. Library preparation was performed with the Nextera XT DNA Sample Kit (Illumina, San Diego, CA, USA), and DNA sequencing was carried out on a NextSeq 500 platform (Illumina) using 2× 150-bp paired-end runs. Reads were trimmed using fqCleanER v.21.10 (https://gitlab.pasteur.fr/GIPhy/fqCleanER), and assemblies were obtained using SPAdes v.3.14.0 (*4*) and polished with Pilon v.1.23 (*5*).

In Silico Molecular Typing

PCR-serogroups (*6*), multilocus sequence types (MLST) (*7*), core genome MLST (cgMLST) (*8*), and virulence and resistance profiles (*8–14*) were extracted from draft assemblies using BIGSdb-*Lm* (*8*,*15*) and BLASTN algorithm, as described before (*8*).

Minimum spanning trees were obtained from MLST and cgMLST profiles using BioNumerics v.7.6 (Applied-Maths, Sint-Martens-Latem, Belgium). MLST analyses also included 351 publicly available *L. monocytogenes* profiles from neighboring countries, obtained from BIGSdb-*Lm* (http://bigsdb.pasteur.fr/listeria; accessed on 16 February 2023). cgMLST-based dendrograms were built in BioNumerics v.7.6.3 (Applied Maths, Sint-Martens-Latem, Belgium) using categorical differences and the single-linkage clustering method, and visualyzed with iTOL v.5 (*16*).

Data Availability

Sequence data was made publicly available in NCBI/EBI/DDJJ databases (BioProject no. PRJEB20026).

Supplementary References

1. US Food and Drug Administration. Testing methodology for *Listeria* species or *L. monocytogenes* in environmental samples. College Park (MD): The Administratio; 2015.

2. International Organization for Standardization. ISO 11290–1:2017 Microbiology of the food chain—Horizontal method for the detection and enumeration of *Listeria monocytogenes* and of *Listeria* spp., 2nd edition. Geneva: The Organization; 2017.

3. Thouvenot P, Vales G, Bracq-Dieye H, Tessaud-Rita N, Maury MM, Moura A, et al. MALDI-TOF mass spectrometry-based identification of *Listeria* species in surveillance: A prospective study. J Microbiol Methods. 2018;144:29–32. PubMed https://doi.org/10.1016/j.mimet.2017.10.009

4. Bankevich A, Nurk S, Antipov D, Gurevich AA, Dvorkin M, Kulikov AS, et al. SPAdes: a new genome assembly algorithm and its applications to single-cell sequencing. J Comput Biol. 2012;19:455–77. PubMed https://doi.org/10.1089/cmb.2012.0021

5. Walker BJ, Abeel T, Shea T, Priest M, Abouelliel A, Sakthikumar S, et al. Pilon: an integrated tool for comprehensive microbial variant detection and genome assembly improvement. PLoS One. 2014;9:e112963. PubMed https://doi.org/10.1371/journal.pone.0112963

6. Doumith M, Buchrieser C, Glaser P, Jacquet C, Martin P. Differentiation of the major *Listeria monocytogenes* serovars by multiplex PCR. J Clin Microbiol. 2004;42:3819–22. PubMed https://doi.org/10.1128/JCM.42.8.3819-3822.2004

7. Ragon M, Wirth T, Hollandt F, Lavenir R, Lecuit M, Le Monnier A, et al. A new perspective on *Listeria monocytogenes* evolution. PLoS Pathog. 2008;4:e1000146. PubMed https://doi.org/10.1371/journal.ppat.1000146

8. Moura A, Criscuolo A, Pouseele H, Maury MM, Leclercq A, Tarr C, et al. Whole genome-based population biology and epidemiological surveillance of *Listeria monocytogenes.* Nat Microbiol. 2016;2:16185. PubMed https://doi.org/10.1038/nmicrobiol.2016.185

9. Ryan S, Begley M, Hill C, Gahan CG. A five-gene stress survival islet (SSI-1) that contributes to the growth of *Listeria monocytogenes* in suboptimal conditions. J Appl Microbiol. 2010;109:984–95. PubMed https://doi.org/10.1111/j.1365-2672.2010.04726.x

10. Palma F, Brauge T, Radomski N, Mallet L, Felten A, Mistou MY, et al. Dynamics of mobile genetic elements of *Listeria monocytogenes* persisting in ready-to-eat seafood processing plants in France. BMC Genomics. 2020;21:130. PubMed https://doi.org/10.1186/s12864-020-6544-x

11. Elhanafi D, Dutta V, Kathariou S. Genetic characterization of plasmid-associated benzalkonium chloride resistance determinants in a *Listeria monocytogenes* strain from the 1998–1999 outbreak. Appl Environ Microbiol. 2010;76:8231–8. PubMed https://doi.org/10.1128/AEM.02056-10

12. Mereghetti L, Quentin R, Marquet-Van Der Mee N, Audurier A. Low sensitivity of *Listeria monocytogenes* to quaternary ammonium compounds. Appl Environ Microbiol. 2000;66:5083–6. PubMed https://doi.org/10.1128/AEM.66.11.5083-5086.2000

13. Lee S, Ward TJ, Jima DD, Parsons C, Kathariou S. The arsenic resistance-associated *Listeria* genomic island LGI2 exhibits sequence and integration site diversity and a propensity for three *Listeria monocytogenes* clones with enhanced virulence. Appl Environ Microbiol. 2017;83:e01189-17. PubMed https://doi.org/10.1128/AEM.01189-17

14. Harter E, Wagner EM, Zaiser A, Halecker S, Wagner M, Rychli K. Stress survival islet 2, predominantly present in *Listeria monocytogenes* strains of sequence type 121, is involved in the alkaline and oxidative stress responses. Appl Environ Microbiol. 2017;83:e00827-17. PubMed https://doi.org/10.1128/AEM.00827-17

15. Jolley KA, Maiden MCJ. BIGSdb: scalable analysis of bacterial genome variation at the population level. BMC Bioinformatics. 2010;11:595. PubMed https://doi.org/10.1186/1471-2105-11-595

16. Letunic I, Bork P. Interactive Tree Of Life (iTOL) v5: an online tool for phylogenetic tree display and annotation. Nucleic Acids Res. 2021;49(W1):W293–6. PubMed https://doi.org/10.1093/nar/gkab301

17. Moura A, Lefrancq N, Wirth T, Leclercq A, Borges V, Gilpin B, et al.; Listeria CC1 Study Group. Emergence and global spread of *Listeria monocytogenes* main clinical clonal complex. Sci Adv. 2021;7:eabj9805. PubMed https://doi.org/10.1126/sciadv.abj9805

18. Moura A, Tourdjman M, Leclercq A, Hamelin E, Laurent E, Fredriksen N, et al. Real-time whole-genome sequencing for surveillance of *Listeria monocytogenes*, France. Emerg Infect Dis. 2017;23:1462–70. PubMed https://doi.org/10.3201/eid2309.170336

19. Halbedel S, Prager R, Fuchs S, Trost E, Werner G, Flieger A. Whole-genome sequencing of recent *Listeria monocytogenes* isolates from Germany reveals population structure and disease clusters. J Clin Microbiol. 2018;56:e00119-18. PubMed https://doi.org/10.1128/JCM.00119-18

Appendix Table 1. Isolate metadata and genome metrics of *Listeria monocytogenes* sequenced in this study (n = 92).

Isolate no.	No. bases after filtering	Coverage	No. contigs	Total length, bp	N50, bp	% GC	% cgMLST loci tagged	Source type	Sample type	Geographic location	Isolation year	Serogroup	Lineage	Clonal complex, MLST	Sublineage, cgMLST	cgMLST type	BIGSdb ID
CLIP 2017/00419	2.40E+08	83	38	3.01E+06	1.94E+05	37.8	99.7	F	Sausage	San José	2016	IVb	I	CC1	SL1	CT2786	ID102753
CLIP 2019/02752	3.82E+08	132	30	2.99E+06	2.62E+05	37.82	99.8	F	Cheese	Vara Blanca	2019	IVb	I	CC1	SL1	CT333	ID102785
CLIP 2019/02623	4.20E+08	145	43	3.04E+06	1.92E+05	37.8	99.9	H	CSF	San José	2018	IVb	I	CC1	SL1	CT333	ID102774
CLIP 2019/02615	5.11E+08	176	38	3.02E+06	2.55E+05	37.81	99.9	F	Cheese	San José	2019	IVb	I	CC1	SL1	CT6042	ID102766
CLIP 2017/00404	3.05E+08	105	58	3.06E+06	1.43E+05	37.82	99.9	F	Cheese	ND	2011	IVb	I	CC2	SL2	CT2715	ID102739
CLIP 2017/00409	2.52E+08	87	110	3.05E+06	7.45E+04	37.85	99.3	F	Cheese	Cartago	2013	IVb	I	CC2	SL2	CT2715	ID102744
CLIP 2017/00417	5.93E+08	205	49	3.01E+06	1.39E+05	37.88	99.8	F	Sausage	San José	2016	IVb	I	CC2	SL2	CT2715	ID102751
CLIP 2019/02742	5.20E+08	179	47	3.17E+06	1.98E+05	37.7	99.8	F	Cheese	Upala	2017	IVb	I	CC2	SL2	CT2715	ID102775
CLIP 2017/00398	4.34E+08	150	58	3.17E+06	1.50E+05	37.69	99.8	H	Blood	Guanacaste†	2013	IVb	I	CC2	SL2	CT2715	ID102733
CLIP 2017/00399	4.18E+08	144	51	3.16E+06	1.32E+05	37.69	99.8	H	Blood	Limón†	2013	IVb	I	CC2	SL2	CT2715	ID102734
CLIP 2017/00400	3.59E+08	124	40	3.10E+06	2.23E+05	37.75	99.8	H	Blood	Limón†	2013	IVb	I	CC2	SL2	CT2715	ID102735
CLIP 2017/00403	5.40E+08	186	52	3.17E+06	1.39E+05	37.69	99.8	H	CSF	Heredia†	2009	IVb	I	CC2	SL2	CT2715	ID102738
CLIP 2017/00391	4.72E+08	163	51	3.04E+06	1.61E+05	37.84	99.9	H	Blood	Cartago†	2016	IVb	I	CC2	SL2	CT2716	ID102726
CLIP 2017/00405	4.46E+08	154	38	3.05E+06	1.39E+05	37.83	99.9	H	Blood	Limón†	2010	IVb	I	CC2	SL2	CT2717	ID102740
CLIP 2019/02751	4.38E+08	151	34	3.06E+06	2.73E+05	37.84	99.9	F	Cheese	Vara Blanca	2019	IVb	I	CC2	SL2	CT2718	ID102784
CLIP 2019/02753	3.36E+08	116	29	3.06E+06	2.72E+05	37.84	99.9	F	Cheese	San Ramón	2019	IVb	I	CC2	SL2	CT2718	ID102786
CLIP 2019/02754	3.03E+08	105	26	2.97E+06	3.33E+05	37.9	99.9	F	Cheese	San Ramón	2019	IVb	I	CC2	SL2	CT2718	ID102787
CLIP 2019/02756	2.54E+08	87	58	3.01E+06	1.44E+05	37.88	99.8	F	Cheese	Zarcero	2019	IVb	I	CC2	SL2	CT2718	ID102789
CLIP 2017/00396	5.98E+08	206	51	3.01E+06	1.41E+05	37.88	99.9	H	CSF	San José	2016	IVb	I	CC2	SL2	CT2718	ID102731
CLIP 2017/00407	4.45E+08	153	58	3.06E+06	1.52E+05	37.83	99.9	H	CSF	Cartago†	2010	IVb	I	CC2	SL2	CT2719	ID102742
CLIP 2017/00390	4.06E+08	140	42	3.04E+06	2.02E+05	37.81	99.9	H	CSF	Alajuela†	2016	IVb	I	CC2	SL2	CT2720	ID102725
CLIP 2017/00392	5.23E+08	180	59	3.03E+06	1.52E+05	37.81	99.8	H	Blood	Alajuela†	2013	IVb	I	CC2	SL2	CT2721	ID102727
CLIP 2017/00401	3.46E+08	119	38	2.96E+06	1.50E+05	37.85	99.8	F	Mushroom	ND	2011	IVb	I	CC2	SL2	CT2762	ID102736
CLIP 2017/00402	2.98E+08	103	42	2.99E+06	1.38E+05	37.81	99.8	F	Mushroom	ND	2011	IVb	I	CC2	SL2	CT2762	ID102737
CLIP 2017/00426	2.63E+08	91	55	3.04E+06	1.87E+05	37.84	99.9	F	Cheese	San José†	2013	IVb	I	CC2	SL2	CT2779	ID102760
CLIP 2017/00420	1.59E+08	55	87	3.05E+06	9.85E+04	37.83	99.9	F	Sausage	San José	2016	IVb	I	CC2	SL2	CT2780	ID102754
CLIP 2017/00421	2.61E+08	90	59	3.05E+06	1.53E+05	37.83	100	F	Sausage	San José	2015	IVb	I	CC2	SL2	CT2780	ID102755
CLIP 2019/02622	3.75E+08	129	48	3.01E+06	2.38E+05	37.86	99.9	H	Blood	San José	2018	IVb	I	CC2	SL2	CT2780	ID102773
CLIP 2017/00413	5.63E+08	194	61	3.08E+06	1.29E+05	37.79	99.9	F	Cheese	San José†	2013	IVb	I	CC2	SL2	CT2787	ID102747
CLIP 2019/02613	4.08E+08	141	41	3.00E+06	1.73E+05	37.86	99.9	F	Cheese	San José	2019	IVb	I	CC2	SL2	CT6040	ID102764
CLIP 2019/02610	4.27E+08	147	67	3.05E+06	1.45E+05	37.79	99.9	F	Cheese	San José	2019	IVb	I	CC2	SL2	CT6041	ID102761
CLIP 2019/02612	3.79E+08	131	62	3.11E+06	1.61E+05	37.86	99.9	F	Cheese	San José	2019	IVb	I	CC2	SL2	CT6041	ID102763
CLIP 2019/02616	3.38E+08	117	65	3.05E+06	1.11E+05	37.79	99.8	F	Cheese	San José	2019	IVb	I	CC2	SL2	CT6041	ID102767
CLIP 2019/02618	3.65E+08	126	82	3.05E+06	1.10E+05	37.79	99.9	F	Cheese	San José	2019	IVb	I	CC2	SL2	CT6041	ID102769
CLIP 2019/02750	4.94E+08	170	50	3.05E+06	1.98E+05	37.79	99.9	F	Cheese	Turrialba	2018	IVb	I	CC2	SL2	CT6041	ID102783
CLIP 2019/02621	5.80E+08	200	51	3.08E+06	1.61E+05	37.84	99.9	H	Blood	San José	2018	IVb	I	CC2	SL2	CT6045	ID102772
CLIP 2019/02611	3.92E+08	135	76	3.09E+06	1.98E+05	37.8	99.9	F	Cheese	San José	2019	IVb	I	CC2	SL2	CT6046	ID102762
CLIP 2019/02617	3.55E+08	122	65	3.03E+06	1.26E+05	37.84	99.9	F	Cheese	San José	2019	IVb	I	CC2	SL2	CT6047	ID102768
CLIP 2019/02745	3.90E+08	135	35	2.97E+06	2.40E+05	37.91	99.9	F	Cheese	Turrialba	2018	IVb	I	CC2	SL2	CT6063	ID102778
CLIP 2019/02748	4.82E+08	166	43	3.04E+06	2.71E+05	37.85	99.9	F	Cheese	Turrialba	2018	IVb	I	CC2	SL2	CT6065	ID102781
CLIP 2019/02757	3.04E+08	105	68	3.10E+06	2.39E+05	37.79	99.7	F	Cheese	San Isidro	2019	IVb	I	CC2	SL2	CT6066	ID102790
CLIP 2019/02744	4.94E+08	170	51	3.04E+06	2.38E+05	37.85	99.8	F	Cheese	Capellades	2018	IVb	I	CC2	SL2	CT6067	ID102777
CLIP 2019/02749	4.85E+08	167	40	3.04E+06	1.59E+05	37.85	99.9	F	Cheese	Santa Cruz	2018	IVb	I	CC2	SL2	CT6069	ID102782
CLIP 2019/02747	4.08E+08	141	74	3.07E+06	1.98E+05	37.83	99.9	F	Cheese	Turrialba	2018	IVb	I	CC2	SL2	CT6070	ID102780
CLIP 2019/02764	7.56E+08	261	84	3.16E+06	3.21E+05	37.76	99.8	F	Raw milk	Turrialba	2019	IVb	I	CC2	SL2	CT6072	ID102797
CLIP 2019/02765	7.81E+08	269	59	3.12E+06	2.06E+05	37.79	99.9	F	Cheese	Turrialba	2019	IVb	I	CC2	SL2	CT6072	ID102798

Isolate no.	No. bases after filtering	Coverage	No. contigs	Total length, bp	N50, bp	% GC	% cgMLST loci tagged	Source type	Sample type	Geographic location	Isolation year	Serogroup	Lineage	Clonal complex, MLST	Sublineage, cgMLST	cgMLST type	BIGSdb ID
CLIP 2019/02759	1.24E+09	426	120	3.11E+06	3.60E+05	38	99.9	PE	Drain	Turrialba	2019	IVb	I	CC2	SL2	CT6072	ID102792
CLIP 2019/02760	7.47E+08	258	47	3.12E+06	3.60E+05	37.77	99.9	PE	Tank	Turrialba	2019	IVb	I	CC2	SL2	CT6072	ID102793
CLIP 2019/02761	7.06E+08	243	62	3.15E+06	3.63E+05	37.81	99.9	PE	Cooling chamber	Turrialba	2019	IVb	I	CC2	SL2	CT6072	ID102794
CLIP 2017/00372	1.76E+08	61	90	3.12E+06	9.53E+04	37.69	99.9	PE	Soil	Cartago†	2016	IVb	I	CC2	SL2	CT6116	ID102721
CLIP 2017/00424	5.53E+08	191	32	3.04E+06	1.98E+05	37.81	99.9	F	Cheese	Heredia	2013	IVb	I	CC2	SL2	CT6117	ID102758
CLIP 2019/02755	3.66E+08	126	37	3.04E+06	3.55E+05	37.84	99.9	F	Cheese	Zarcero	2019	IVb	I	CC2	SL2	CT6118	ID102788
CLIP 2017/00412	2.08E+08	72	60	3.06E+06	1.35E+05	37.8	99.8	F	Cheese	Cartago	2013	IVb	I	CC2	SL2	CT6119	ID102746
CLIP 2017/00411	2.77E+08	96	57	3.08E+06	2.81E+05	37.8	99.8	F	Cheese	Cartago	2013	IVb	I	CC2	SL2	CT6120	ID102745
CLIP 2017/00415	2.78E+08	96	51	3.04E+06	1.55E+05	37.84	99.9	F	Cheese	Cartago†	2013	IVb	I	CC2	SL2	CT6120	ID102749
CLIP 2017/00418	2.74E+08	94	48	3.04E+06	1.50E+05	37.84	99.9	F	Cheese	San José	2013	IVb	I	CC2	SL2	CT6120	ID102752
CLIP 2017/00423	2.62E+08	90	63	3.08E+06	1.39E+05	37.8	99.9	F	Cheese	Cartago	2013	IVb	I	CC2	SL2	CT6120	ID102757
CLIP 2019/02614	2.43E+08	84	90	3.08E+06	9.05E+04	37.81	99.4	F	Cheese	San José	2019	IVb	I	CC2	SL2	CT6120	ID102765
CLIP 2019/02746	3.45E+08	119	59	3.08E+06	1.61E+05	37.81	99.8	F	Cheese	Turrialba	2018	IVb	I	CC2	SL2	CT6120	ID102779
CLIP 2019/02762	6.86E+08	236	475	3.27E+06	2.73E+05	39.67	99.8	F	Cheese	Turrialba	2019	IVb	I	CC2	SL2	CT6120	ID102795
CLIP 2019/02763	9.18E+08	317	136	3.12E+06	3.58E+05	38.07	99.9	F	Raw milk	Turrialba	2019	IVb	I	CC2	SL2	CT6120	ID102796
CLIP 2017/00393	4.76E+08	164	59	3.08E+06	1.50E+05	37.8	99.9	H	Blood	Cartago†	2016	IVb	I	CC2	SL2	CT6120	ID102728
CLIP 2017/00408	4.09E+08	141	66	3.08E+06	1.36E+05	37.81	99.9	H	CSF	Cartago†	2010	IVb	I	CC2	SL2	CT6120	ID102743
CLIP 2017/00406	6.63E+08	228	60	3.07E+06	1.38E+05	37.79	99.9	H	CSF	Cartago†	2015	IVb	I	CC2	SL2	CT6121	ID102741
CLIP 2017/00389	3.43E+08	118	41	3.05E+06	1.39E+05	37.83	99.9	F	Meat	ND	2016	IVb	I	CC2	SL2	CT6122	ID102724
CLIP 2019/02758	8.26E+08	285	31	2.91E+06	4.33E+05	37.91	99.8	F	Cheese	Naranjo	2019	IVb	I	CC217	SL217	CT222	ID102791
CLIP 2017/00395	3.03E+08	104	41	3.00E+06	1.95E+05	37.79	99.9	F	Sausage	ND	2016	IVb	I	CC6	SL6	CT2761	ID102730
CLIP 2017/00383	3.27E+08	113	59	3.03E+06	1.55E+05	37.82	99.9	F	Shrimp	San José†	2016	IIb-v1	I	ST1079	SL1079	CT1669	ID102722
CLIP 2017/00357	3.37E+08	116	53	3.14E+06	1.58E+05	37.75	99.8	F	Ground beef	San José†	2016	IIb	I	CC3	SL3	CT1674	ID102707
CLIP 2017/00366	3.20E+08	110	51	3.10E+06	1.97E+05	37.79	99.9	PE	Drain	San José	2016	IIb	I	CC3	SL3	CT1674	ID102716
CLIP 2017/00358	2.38E+08	82	45	3.05E+06	1.34E+05	37.81	99.8	F	Chicken wings	San José	2016	IIb	I	CC3	SL3	CT2730	ID102708
CLIP 2017/00360	3.67E+08	127	43	3.05E+06	1.86E+05	37.81	99.8	F	Pork	Heredia	2016	IIb	I	CC3	SL3	CT2730	ID102710
CLIP 2017/00361	5.29E+08	183	44	3.05E+06	1.87E+05	37.81	99.8	F	Pork	San José†	2016	IIb	I	CC3	SL3	CT2730	ID102711
CLIP 2017/00363	4.02E+08	139	51	3.05E+06	1.42E+05	37.81	99.7	F	Cheese	San José	2016	IIb	I	CC3	SL3	CT2730	ID102713
CLIP 2017/00364	4.97E+08	171	44	3.05E+06	1.69E+05	37.81	99.7	F	Ground beef	San José†	2016	IIb	I	CC3	SL3	CT2730	ID102714
CLIP 2017/00365	2.15E+08	74	43	3.05E+06	1.97E+05	37.81	99.8	F	Tilapia	Guanacaste†	2016	IIb	I	CC3	SL3	CT2730	ID102715
CLIP 2017/00359	3.82E+08	132	40	3.05E+06	3.71E+05	37.81	99.8	PE	Drain	Alajuela	2016	IIb	I	CC3	SL3	CT2730	ID102709
CLIP 2017/00370	3.05E+08	105	40	3.05E+06	1.97E+05	37.81	99.8	PE	Drain	San José	2016	IIb	I	CC3	SL3	CT2730	ID102719
CLIP 2017/00371	5.02E+08	173	42	3.05E+06	2.36E+05	37.81	99.7	PE	Drain	San José	2016	IIb	I	CC3	SL3	CT2730	ID102720
CLIP 2017/00422	2.31E+08	80	39	3.04E+06	2.53E+05	37.92	99.7	F	Cheese	Cartago	2013	IIb	I	CC3	SL3	CT2781	ID102756
CLIP 2017/00425	2.84E+08	98	39	3.04E+06	1.97E+05	37.93	99.7	F	Cheese	Cartago	2013	IIb	I	CC3	SL3	CT2781	ID102759
CLIP 2017/00367	2.62E+08	90	79	3.11E+06	2.36E+05	37.79	99.9	PE	Cooling chamber	San José	2016	IIb	I	CC5	SL5	CT2783	ID102717
CLIP 2017/00362	4.62E+08	159	74	3.11E+06	1.35E+05	37.77	99.9	F	Tilapia	San José	2016	IIb	I	CC5	SL5	CT2793	ID102712
CLIP 2017/00368	5.08E+08	175	59	3.10E+06	1.53E+05	37.79	99.9	F	Beef	Heredia†	2016	IIb	I	CC5	SL5	CT2793	ID102718
CLIP 2017/00414	2.13E+08	73	77	2.93E+06	1.20E+05	37.86	99.4	F	Cheese	Heredia†	2013	IIb	I	CC506	SL506	CT2776	ID102748
CLIP 2019/02619	3.78E+08	130	39	2.97E+06	3.04E+05	37.88	99.9	F	Chorizo	Heredia	2019	IIb	I	CC87	SL87	CT6044	ID102770
CLIP 2017/00416	2.36E+08	81	38	2.92E+06	1.99E+05	37.91	99.9	F	Sausage	San José	2016	IIb	I	CC87	SL87	CT65	ID102750
CLIP 2019/02620	3.96E+08	137	39	2.97E+06	2.09E+05	37.88	99.9	F	Chorizo	San José	2019	IIb	I	CC87	SL87	CT65	ID102771
CLIP 2017/00397	4.04E+08	139	28	3.09E+06	3.73E+05	37.87	100	F	Sausage	ND	2016	IIc	II	CC9	SL9	CT13239	ID102732
CLIP 2017/00388	2.98E+08	103	44	3.04E+06	2.02E+05	37.8	100	F	Pork	ND	2016	IIc	II	CC9	SL9	CT1668	ID102723

Isolate no.	No. bases after filtering	Coverage	No. contigs	Total length, bp	N50, bp	% GC	% cgMLST loci tagged	Source type	Sample type	Geographic location	Isolation year	Serogroup	Lineage	Clonal complex, MLST	Sublineage, cgMLST	cgMLST type	BIGSdb ID
CLIP 2017/00394	3.41E+08	117	38	3.09E+06	2.01E+05	37.79	100	F	Frozen vegetables	ND	2016	IIa	II	CC121	SL121	CT909	ID102729
CLIP 2019/02743	3.86E+08	133	18	2.83E+06	3.82E+05	37.93	99.9	F	Cheese	Turrialba	2018	IIa	II	CC19	SL378	CT6064	ID102776

*BIGSdb, Bacterial Isolate Genome Sequence Database (*17*); cgMLST, core-genome multilocus sequence typing; F, food; H, human; ID, identification; MLST, multilocus sequence typing; ND, not done; PE, production environment.
†Geographic information available only at the level of the province.

Appendix Table 2. Previously reported cgMLST types detected in this study (cutoff of 7 or less allelic differences out of 1748 cgMLST loci, Institut Pasteur scheme)

cgMLST Type (CC, serogroup)	Source (item)		Studies from other countries	Source lab	NCBI/EBI/DDJJ accession no.	Reference no.
	This study	Other studies				
Lineage I						
L1-SL217-ST217-CT222 (CC217, IVb)	F (dairy)	H, F (salad)	US	CDC	SRR1021894, SRR1027089	(*8*)
L1-SL1-ST1-CT333 (CC1, IVb)	H, F (dairy)	H	US	CDC	SRR1043171, SRR7057542	(*8*,*17*)
L1-SL87-ST847-CT65 (CC87, IIb)	F (meat)	F (avocado)	MX	FDA	SRR975360	(*8*)
Lineage II						
L2-SL121-ST121-CT909 (CC121, IIa)	F (frozen vegetables)	H, F, PE, FE, A	CL, DK, FR, DE, LV, NO, PL, NL, UK, US	KMAHVH, ANSES, ECDC, IP, UWLMO, UKHSA, FDA, RKI	ERR1304231, ERR1738638, ERR1738650, ERR2522041, ERR2522263, ERR2522276, ERR2522284, ERR2522292, ERR2522294, ERR2522295, ERR2522297, ERR2522312, ERR2522337, ERR2522338, ERR2522353, ERR2522363, ERR2522371, ERR2522810, ERR3040059, ERR3040061, ERR3040067, ERR3040072, ERR3040073, ERR3040075, ERR3040076, ERR3040078, ERR3040083, ERR3040104, ERR4176463, ERR4176497, ERR4176536, ERR4176606, ERR4176666, ERR4176794, ERR4648214, SRR10753542, SRR10753574, SRR10753599, SRR10753600, SRR10753602, SRR11004717, SRR13072765, SRR14783257, SRR15245555, SRR15245949, SRR20053048, SRR2040689, SRR4052010, SRR4052014, SRR4052068, SRR4052071, SRR4052072, SRR4052073, SRR4052161, SRR4124934, SRR4124940, SRR5133499, SRR5318940,	(*8*,*10*,*18*,*19*)

cgMLST Type (CC, serogroup)	Source (item)		Studies from other countries	Source lab	NCBI/EBI/DDJJ accession no.	Reference no.
	This study	Other studies				
					SRR5526029, SRR5526031, SRR5526035, SRR5526085, SRR5526092, SRR5526094, SRR5526097, SRR5526099, SRR5526101, SRR5526105, SRR5526120, SRR5526126, SRR5526128, SRR5526130, SRR5526135, SRR5526137, SRR5526138, SRR5526142, SRR5526145, SRR5526155, SRR5647016, SRR5647027, SRR5647029, SRR5647030, SRR6966182, SRR7403111, SRR7410624, SRR7429735, SRR7429781, SRR7440567, SRR7440606, SRR7440615, SRR7440618, SRR7440621, SRR7440634, SRR7440649, SRR7440688, SRR7440921, SRR7441075, SRR7441146, SRR7441209, SRR7441250, SRR7441287, SRR7547848, SRR7827106, SRR7828078, SRR7839361, SRR7841130, SRR7841155, SRR7841205, SRR7842337, SRR7850202, SRR7850362, SRR7850428, SRR7850452, SRR7866225, SRR7866376, SRR7866514, SRR7866571, SRR7866588, SRR7866636, SRR7866672, SRR7866727, SRR7866750, SRR7866891, SRR7873595, SRR7873692, SRR7873717,	

cgMLST Type (CC, serogroup)	Source (item) This study	Source (item) Other studies	Studies from other countries	Source lab	NCBI/EBI/DDJJ accession no.	Reference no.
					SRR7873744, SRR8235628, SRR8239064, SRR8239220, SRR8239239, SRR8655304, SRR8979699, SRR9004322, SRR9010038	

*A, animal; ANSES, Agence Nationale de Sécurité Sanitaire de L'Alimentation, de L'Environnement et du Travail, FR; CC, clonal complex; CDC, US Centers for Disease Control and Prevention; CL, Chile; DE, Germany; DK, Denmark; ECDC, European Centre for Disease Prevention and Control; F, food; FDA, US Food and Drug Administration;FE, farm environment; FR, France; H, human; IP, Institut Paster, FR; KMAHVH, Klinisk Mikrobiologisk Afdeling, DK; LV, Latvia; MX, Mexico; NO, Norway; PE, food production environment; PL, Poland; RKI, Robert Koch Institut, DE; UK, United Kingdom; UKHSA, UK Health Security Agency, UK; US, United States; UWLMO, University of Warmia and Mazury in Olsztyn, PL.

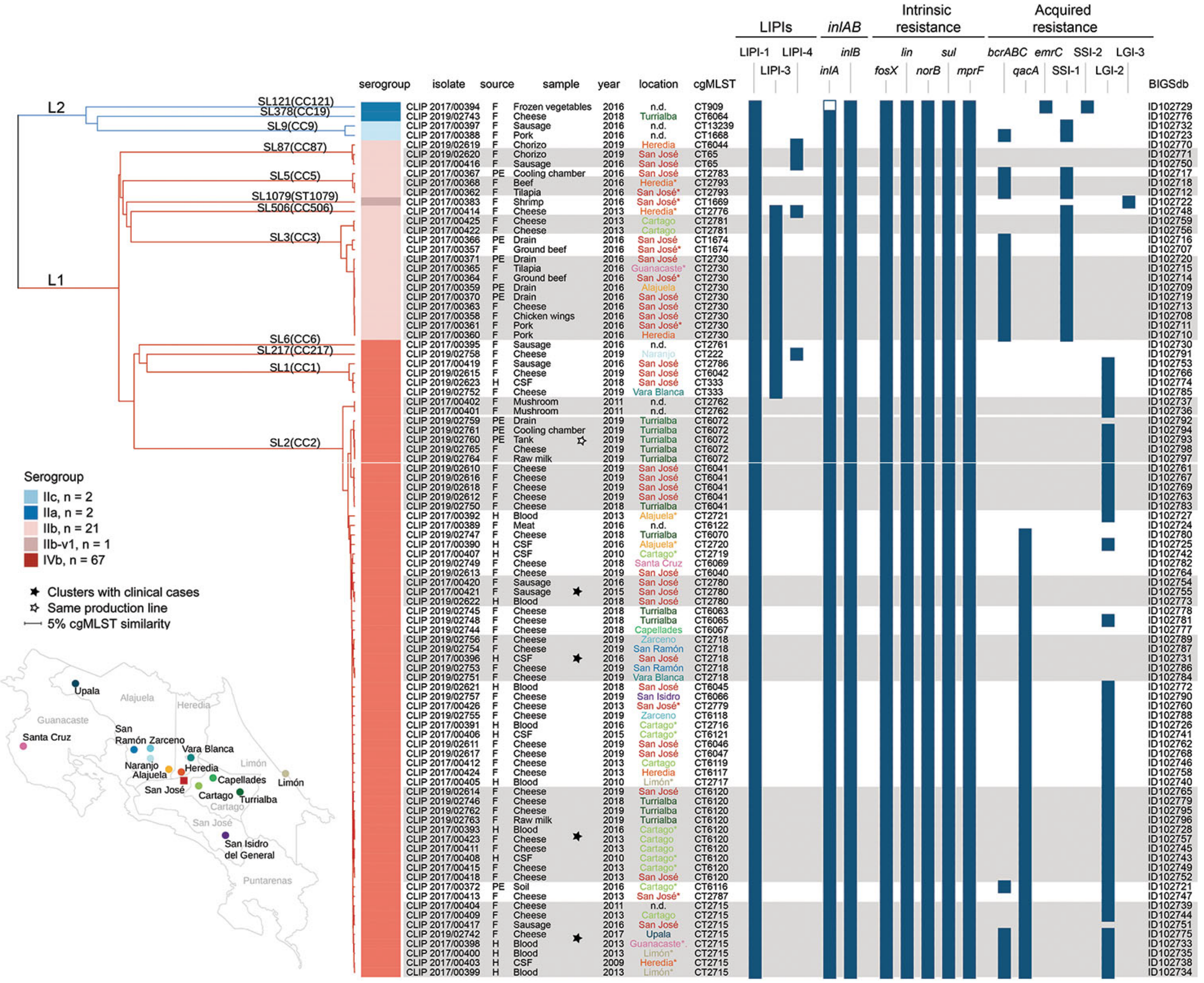

Appendix Figure 1. Single linkage dendrogram of 92 isolates generated for genome-based characterization of *Listeria monocytogenes*, Costa Rica. Dendrogram based on cgMLST allelic profiles (1,748-locus scheme). Branches are colored according to lineages: L1, red; L2, blue. Branches are labeled according to lineages, sublineages, and clonal complexes. Information on isolates' name, serogroup, source, sample type, cgMLST type, year of isolation, location, and BIGSdb IDs are provided in the columns. Colors in location column correspond to dots on map. Gray bars indicate clusters of isolates

with <7 allelic differences out of 1,748 cgMLST loci. presence of selected virulence and resistance genetic traits in each isolate is represented by squared dark blue boxes and empty boxes denote genes with premature stop codons. BIGSdb, Bacterial Isolate Genome Sequence Database (https://bigsdb.pasteur.fr); CC, clonal complex; cgMLST, core-genome multilocus sequence typing; F, food; H, human; ID, identification; L, lineage; LIPI, listeria pathogenicity island; SL, sublineage; ST, sequence type.

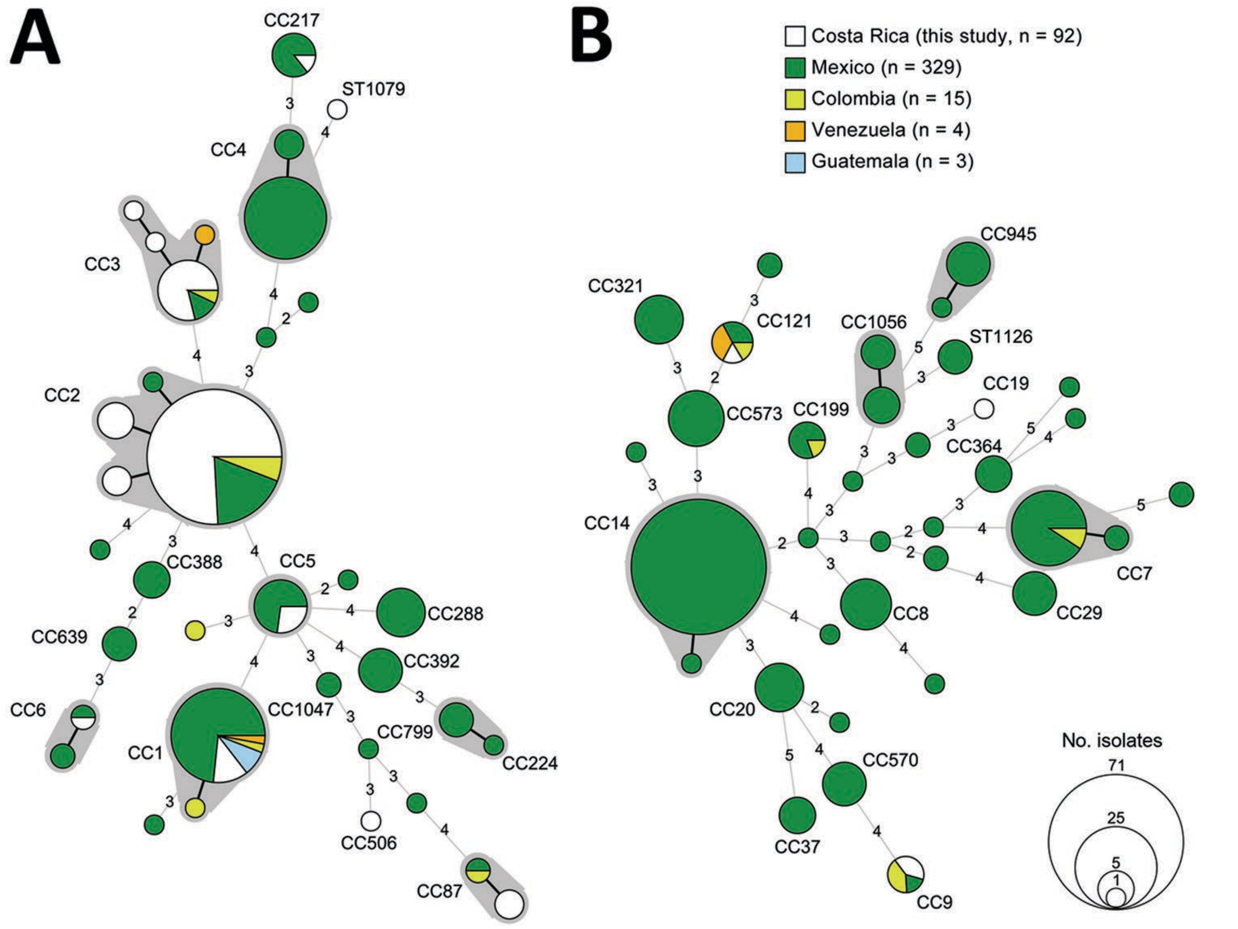

Appendix Figure 2. Minimum spanning tree of 92 *Lysteria monocytogenes* isolates, Costa Rica, 2009–2019. Trees are based on cgMLST allelic profiles (7-locus scheme). A) Lineage I isolates; B) lineage 2 isolates. Publicly available *L. monocytogenes* isolates (n = 351) from neighboring countries in the Caribbean region were also included. Circles represent different profiles and sizes are proportional to the number of isolates within. Branch lengths are proportional to the allelic differences between the profiles which are indicated in the branches. For simplicity, allelic differences of 1 are omitted and represented by thicker branch lines. Clonal complexes with >1 profile are surrounded by gray shading and labeled if detected in this study or if they contain >5 isolates. CC, clonal complex; cgMLST, core-genome multilocus sequence typing; ST, sequence type.

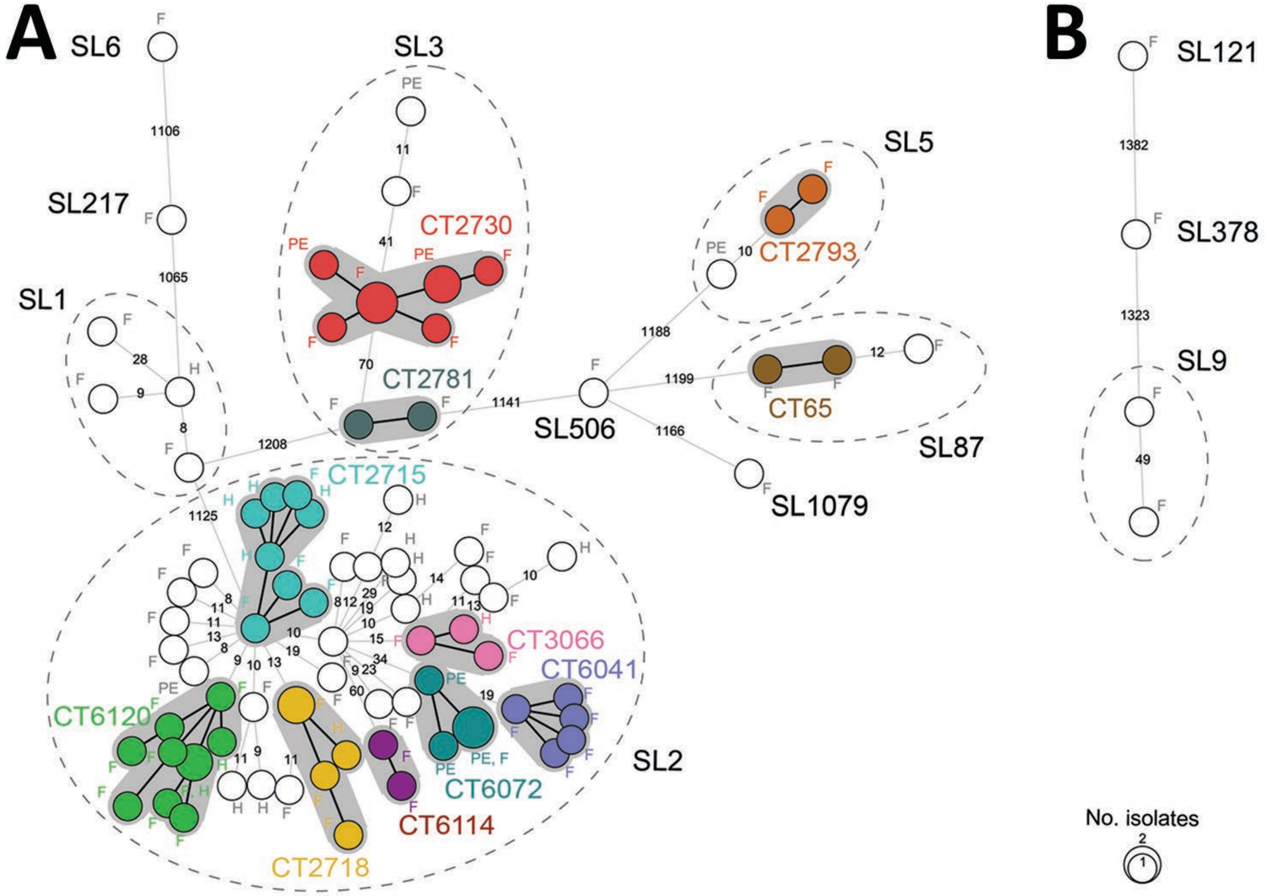

Appendix Figure 3. Minimum spanning tree of 92 *Lysteria monocytogenes* isolates, Costa Rica, 2009–2019. Trees are based on cgMLST allelic profiles (1,748-locus scheme). A) Lineage I isolates; B) lineage 2 isolates. Circles represent different profiles and sizes are proportional to the number of isolates within. Labels next to circles indicate the source of isolates. Branch lengths are proportional in logarithmic scale to allelic differences between profiles, which are also indicated in the branches. For simplicity, allelic differences of $\leq$7 are omitted and represented by thicker branch lines. Clusters with >1 profile are highlighted in colors; labels correspond with cgMLST type and are delimitated by gray shadows. Dashed ellipses delimitate sublineages with >1 isolate and labeled with corresponding sublineage. cgMLST, core-genome multilocus sequence typing; CT, cgMLST type; F, food; H, human, PE; production environment; SL, sublineage.
